# A prospective multicenter study on bladder cancer: the COBLAnCE cohort

**DOI:** 10.1186/s12885-016-2877-x

**Published:** 2016-11-03

**Authors:** Simone Benhamou, Julia Bonastre, Karine Groussard, François Radvanyi, Yves Allory, Thierry Lebret

**Affiliations:** 1 INSERM, UMR 946, Genetic Variation and Human Diseases Unit, 27 rue Juliette Dodu, 75010 Paris, France; 2 Université Paris Diderot, Sorbonne Paris Cité, Institut Universitaire d’Hématologie, Paris, France; 3 Gustave Roussy, Service de biostatistique et d’épidémiologie, Villejuif, France; 4 CESP Centre for Research in Epidemiology and Population Health, INSERM U1018, University Paris-Sud, UVSQ, Université Paris-Saclay, Villejuif, France; 5 CNRS, UMR144, Equipe Labellisée par la Ligue Nationale contre le Cancer, Paris, France; 6 Institut Curie, PSL Research University, Paris, France; 7 Université Paris-Est, Créteil, France; 8 INSERM, UMR 955, Créteil, France; 9 Département de pathologie, APHP, Hôpital Henri-Mondor, Créteil, France; 10 Service d’urologie et de transplantation rénale, Hôpital Foch, Suresnes, France; 11 UFR des sciences de la santé Simone Veil, Université de Versailles-Saint-Quentin-en-Yvelines, Suresnes, France

**Keywords:** Bladder cancer, Cohort study, Biobanks, Gene-environment interactions, Molecular classification, Biomarkers, Healthcare resource use, Quality of life, Outcomes

## Abstract

**Background:**

Bladder cancer is a very heterogeneous disease as regards natural history. Environmental exposures, constitutional genetic and/or epigenetic background may affect not only the likelihood of bladder tumor occurrence, but also the histologic type of cancer and its outcome. Currently, only a few data are available to study the prognostic role of genetic and environmental factors. Likewise, data on the economic burden of bladder cancer and the longitudinal impact of the disease and the treatments on patient quality of life are scarce.

**Methods:**

COBLAnCE is a large French-based clinical cohort study on bladder cancer. Newly diagnosed patients are enrolled prospectively in 12 public hospitals and 5 private for profits hospitals. The target sample size is 2,000 patients. All patients are to be followed for 6 years. Information on patient characteristics and lifestyle is collected during a face-to-face interview at enrollment. Clinical information on disease presentation, diagnosis, and treatment is extracted from medical records for the primary tumor and for all subsequent local and distant recurrences. Quality of life and resource use is collected at recruitment and during follow-up. In parallel, 4 types of biological samples (blood, tumor tissue, urine and nail) are collected, at baseline and during follow-up. DNA, RNA and PBMLs are extracted from blood samples, DNA and RNA from stabilized urine, proteins from frozen urine, DNA, RNA and proteins from frozen tumor tissues, and DNA and RNA from formalin-fixed paraffin-embedded tumor tissues. All derived products are stored at −80 °C or in liquid nitrogen. Main endpoints are gene-environment interactions, molecular classification, biomarker discovery, therapeutic innovation, treatment patterns, healthcare resource use, bladder cancer outcomes and quality of life.

**Discussion:**

The COBLAnCE cohort will provide considerable insight into the biology of bladder cancer and the mechanisms through which genetic and environmental factors may influence the prognosis. It may allow the discovery of emerging biomarkers. Finally, economic data will be useful for future cost-effectiveness studies of immunotherapy drugs or other therapeutics in bladder cancer.

## Background

In Europe, bladder cancer is the second most common malignancy of the urinary tract, after prostate cancer, in men [1]. In 2012, the estimated incidence of bladder cancer was 151 300, of which 118 400 were diagnosed in men and 32 900 were diagnosed in women [1]. In men, bladder cancer was the fourth most commonly diagnosed cancer and accounted for 4.0 % of all cancer-related deaths [1]. Approximately ¾ of bladder cancers occur at the age of 65 years or more [1]. Main risk factors include tobacco smoking and some occupational exposures [2]. Genetic factors could also contribute to bladder cancer development. The risk of bladder cancer is 2-fold higher in first-degree relatives of bladder cancer patients. Common polymorphisms in two carcinogen detoxification genes, *N-acetyltransferase 2* (*NAT2*) and *glutathione-S-transferase M1* (*GSTM1*), have been consistently associated with bladder cancer risk and several studies suggested that the *NAT2* slow acetylation genotype interacts with smoking intensity [3]. However, additional large investigations of bladder cancer risk, *NAT2* genetic variation and comprehensive assessment of smoking habits, notably flue- or air-cured tobacco, are still needed to further assess this interaction. Recently, genome-wide association studies have identified around ten common susceptibility loci associated with bladder cancer risk [4, 5]. In contrast, little is known on the relationships between genetic markers, behavioral and environmental factors, and bladder cancer outcome.

Bladder cancer is a very heterogeneous disease with a variable natural history. Ninety to 95 % of all bladder cancers are urothelial cell carcinomas. Urothelial carcinomas are classified into two broad categories: non-muscle-invasive bladder cancers (NMIBC) which are restricted to the urothelial cell layers (stage Ta or Tis) or only penetrate the lamina propria (stage T1), and muscle-invasive bladder tumors (MIBC). About 80 % of all newly diagnosed bladder carcinomas present as NMIBC and 20 % as MIBC. The disease is characterized by a wide range of outcomes. At one end of the spectrum, low-grade NMIBC rarely progress to aggressive or metastatic tumors but as many as half will recur [6]. At the other extreme, high-grade NMIBC have a high malignant potential associated with significant progression (20 % of patients develop a MIBC bladder cancer), and 50 % of MIBC are associated with cancer death [7].

In the United States, several studies have shown that bladder cancer represents an economic burden for patients and society [8]. However, few economic data based on patient level data are available in Europe especially regarding the cost-effectiveness of many recent and costly interventions. Given the major differences between healthcare systems, it is important to assess the economic burden of bladder cancer in different jurisdictions [9]. Similarly, good quality data on the longitudinal impact of both the disease and the treatments on the patient quality of life (QoL) are scarce [10]. In particular, there is limited QoL data in metastatic patients and in non-muscle invasive tumors where the rhythm of endoscopic surveillance could be adapted according to the repercussions on the various dimensions of QoL (functional dimension and anxiety).

Overall, environmental exposures, constitutional genetic and/or epigenetic background may affect not only the likelihood of bladder tumor occurrence but also the type of cancer which will occur. More specifically, they may determine the oncogenesis pathway followed by bladder tumorigenesis. Today, there are only very few data available to address these issues, probably because of a lack of samples of patients well enough clinically, epidemiologically and biologically characterized. There is therefore a need for well designed, adequately-powered cohort study to investigate gene-environment interactions, pathological and molecular classification, biomarker discovery, therapeutic innovation, treatment patterns, healthcare resource use, bladder cancer outcomes and quality of life.

## Methods/Design

### Study design

COBLAnCE (a *CO*hort to study *BLA*dder *C*anc*E*r) is a national, non-interventional, prospective cohort of newly diagnosed bladder cancer patients. Twelve French public hospitals (including 9 university institutions) and 5 private for profits hospitals participate in the enrollment. The study protocol has been approved by the French regulatory authorities (data protection authority Commission Nationale de l’Informatique et des Libertés (CNIL) N° DR-2012-441 and biological collections authority N° DC-2011-1486) and a French ethics committee (Comité de Protection des Personnes Ile de France VII (N° CO-12-001). COBLAnCE adapted the Integrated Study on Bladder Cancer (ISBlaC) tools that were kindly provided by Dr Malats (National Cancer Research Centre (CNIO), Madrid, Spain). Each patient enrolled in the study provides a written informed consent. Enrollment started in December 2012.

The study has been designated:

1. To evaluate the association between constitutional DNA polymorphisms, environmental parameters, molecular bladder cancer subtypes and outcomes. The inclusion of features of both the tumor and the host will facilitate the discovery of tumorigenesis mechanisms, drugable targets and diagnostic and prognostic biomarkers.
  We will comprehensively assess interactions between smoking habits, notably flue- or air-cured tobacco, and genetic variants on bladder cancer risk. Such analyses are possible in France where the great majority of cigarette smokers were air-cured tobacco users before the 1970’s. Changes in smoking habits are recorded during the follow-up period and will allow evaluating the impact of continuing smoking after bladder cancer diagnosis on recurrence and survival. More generally, this study will make possible the assessment of the impact of various environmental and occupational exposures on bladder cancer presentation and prognosis, and whether these relationships are modulated by genetic markers.
2. To improve the molecular subtyping of bladder cancer on a large series of tumors collected prospectively integrating transcriptome (both coding and non-coding RNA), proteome, genomic alterations and epigenetic modifications. The identification of homogeneous classes of bladder cancers is a fundamental step towards tailored treatments and more reliable predictive markers of treatment response.
  Beyond the NMIBC / MIBC dichotomy, the huge heterogeneity of bladder cancer requires large number of samples and high throughput technologies to unravel reliable molecular subtypes. The specific associations between molecular subtype occurrence and genetic and/or environmental background will be explored.
3. To provide insight into the tumor progression comprehension and to help to identify biomarkers by the sequential sampling of recurrent tumors and urine from individual patients during follow-up.
  Molecular comparison of primary tumor with samples at recurrence and/or progression will allow phylogenetic analysis, phenotypic stability and tumor heterogeneity assessment. The specifically molecular changes associated with clinical steps will be tested as potential driving events and/or prognosis biomarkers.
4. To describe treatment patterns and to assess QoL and direct and indirect costs attributable to bladder cancer from the payer perspective with a long-term follow-up.
  These data will help decisions by all stakeholders and policymakers. It will be of paramount importance in the context of implementation of new diagnostic or therapeutic modalities.

### Patient population

Patients, regardless of age and sex, are eligible to participate in the study if they are diagnosed in one of the participating centers with no history of bladder cancer, with at least one signal (symptoms, imaging results) that is suspected to be of bladder cancer origin and are to undergo transurethral resection or cystectomy in order to obtain a final histologic diagnosis. All histological types are eligible.

### Follow-up schedule

All patients will be followed over a 6-year period as most first recurrence and cases of progression occur during this time scale. Treatment follow-up is based on the European Association of Urology recommendations, i.e. routine visits at 3 months, 6 months, 12 months for high grade tumors including cytology and cystoscopy, and annually thereafter [11]. Information on health status is recorded at each follow-up visit.

### Data collection and data management

Information on patient characteristics and lifestyle is collected during a face-to-face interview at enrollment. Interviews are conducted by trained interviewers at each of the 17 recruiting centers and take approximately 50 min. Clinical information on disease presentation, diagnosis, and treatment is extracted from patient medical records for the primary tumor and for all subsequent local and distant recurrences. Quality of life is collected using self-administered questionnaires at recruitment and at 6 months, 12 months, and then annually if no local recurrence occur, and at 3 months, 6 months 12 months and then annually in case of local recurrence. Two types of questionnaires are used: the EuroQol EQ-5D questionnaire and the EORTC QLQ-C30 core questionnaire as well as additional modules, which have been developed to measure QoL in patients with bladder cancer (BLS 24 and BLM 30). Finally, resource use is collected during the different steps of the management of bladder cancer distinguishing between non-muscle-invasive and muscle-invasive tumors: diagnosis, surgery, adjuvant treatments, surveillance, and treatment of local and distant recurrences. Main data collected are provided in Table 1.

**Table 1:** Data collection

Type of data	Data collected	Data Source
Epidemiology	(1) Sociodemographic characteristics: age, sex, place of birth, educational level, marital status, household income, working situation, history of residences and of occupations. (2) Lifestyle: history of tobacco consumption, dietary habits and physical activity. (3) Medical history and medication use: weight, height, urinary tract infection, hematuria, kidney stones, skin or respiratory allergies, anti-hypercholesteremic and anti-inflammatory drugs, hormonal treatment, personal and family history of cancer	Face to face interview
Disease management	(1) Disease presentation: presence of symptoms (hematuria, pollakuria, dysuria, urgency, hydronephrosis), alteration of health status (2) Procedures before diagnostic resection: urinary cytology, urine culture, abdominal ultrasound, urinary tract fibroscopies, imaging (scanner, MRI) (3) Diagnosis through transurethral resection of the bladder: Hervix or Narrow-Band-Imaging (NBI) fluorescence, number and location of resected tumors, size and aspect of the largest tumor (4) Treatment: for NMIBC intravesical instillations and cystectomy; for MIBC: chemotherapy, radiotherapy, for both NMIBC and MIBC: lymphadenectomy, urethrectomy, nephro-urethrectomy, urinary diversion, blood transfusion, any hospitalization and complications. (5) Outcomes: locoregional and distant recurrences (dates, sites, pathology, treatments, etc.) and death (date and place)	Patient medical records
Pathology	(1) Histological type and subtype (2) Prognosis factors : for NMIBC tumor stage, grade, CIS associated, lymphovascular invasion; for MIBC, TNM stage, surgical margins, lymphovascular invasion, lymph node density (3) Pathological review for all cases at initial diagnosis and recurrence	Patient medical records
Resource use	(1) All hospitalizations: dates, type of facility, service, type of care and diagnosis-related group. (2) Outpatient and community care : Cystoscopies, Imaging (CT, MRI, ultrasound, etc.), urine cytology, urine culture and tumor biomarkers (3) Sick leaves	Patient medical records Self-administered questionnaire
Quality of life	(1) Generic measure: EuroQol EQ-5D-3 L questionnaire (2) Generic measure: EORTC QLQ-C30 questionnaire (3) Specific measures: urinary and bowel symptoms, impairment due to repeated treatments and sexual functioning. - EORTC-BLS24 questionnaire for patients treated with transurethral resection of the bladder - EORTC-BLM30 questionnaire for those who underwent cystectomy	Self-administered questionnaires

Structured and secured electronic Case Report Forms (eCRFs) have been developed for the study. Subjects are assigned a study identification number which identifies their data. Names are removed from the data and the correspondence between the name and the identification number is kept in centers. eCRFs are entered online at each participating center into a central database (‘MACRO’) specifically designed to store all collected data. The database comprises automatic range and logic checks to reduce data entry errors. A data manager identifies missing or inconsistent data and returns queries to centers.

### Sample collection

For each patient, four types of samples (blood, tumor tissue, urine and nail) are collected, at baseline and/or during follow-up, and delivered to three Biological Resources Centers (BRC) (NF S96-900 certification obtained for two BCR and being requested for one BRC) for processing and storage in cryoconservators or freezers which are alarmed and continuously monitored (Table 2).

**Table 2:** Sample collection

Time of collection	Sample	Derived products	Storage
Enrollment	(1) Blood	DNA, RNA, PBMLs	−80 °C and liquid nitrogen
	(2) Stabilized urine Frozen urine	DNA, RNA Proteins	−80 °C −80 °C
	(3) Frozen tumor FFPE	DNA, RNA, proteins DNA, RNA	−80 °C −80 °C
	(4) Nail		Room temperature
12 months after enrollment	(1) Stabilized urine Frozen urine	DNA, RNA Proteins	−80 °C −80 °C
At each recurrence	(1) Frozen tumor FFPE	DNA, RNA, proteins DNA, RNA	−80 °C −80 °C

PBMLs, peripheral blood mononuclear leukocytes; FFPE, formalin-fixed paraffin-embedded

Sampling kits including all tubes/containers required for the study and packaging for the shipment are prepared by the BRC of the Jean Dausset Foundation – Human Polymorphism Study Center (HPSC-BRC) in Paris and sent to participating centers. All contents are pre-labeled with a unique bar code to provide full traceability from the patient inclusion.

#### Blood samples

Blood samples are collected prior to surgery in three tubes (10 ml in one EDTA-containing tube for DNA extraction and isolation of plasma, 6 ml in one citrate-containing tube for isolation of lymphocytes, and 2.5 ml in one PAXgene tube for RNA extraction). The same day samples are shipped by centers to the HPSC-BRC where they are processed at reception (24 to 72 h after blood sampling). Viable lymphocytes are isolated using ‘UNI-SEP Lymphocyte Separation’ tubes (Eurobio) and stored in liquid nitrogen. Aliquots of plasma and buffy-coats isolated from EDTA blood are stored at −80 °C.

DNA is extracted from buffy-coats using a salting out protocol on the Autopure LS (Qiagen). DNA concentrations are measured using ‘PicoGreen dsDNA reagent’ (Life Technologies). DNAs are diluted sequentially using TE10:1 to get concentrations normalized at 100 ng/μL. Integrity of DNA is checked on agarose gel on 20 % of extracted DNAs. Gender is systematically checked on all DNAs extracted using a PCR method [12].

RNA is extracted from PAXgene tubes using PAXgene Blood miRNA Kit (Qiagen) on the QIAcube (Qiagen). RNA concentration is measured on a NanoDrop 2000C (Thermo Scientific) and quality of RNA is evaluated by calculation of the RIN (RNA Integrity Number) on a Bioanalyzer 2100 (Agilent).

#### Tumor samples

Tumor tissue is sampled at first transurethral resection of bladder tumor and transferred to the pathology department for diagnosis according to routine procedures, including formalin fixation and embedding in paraffin (FFPE blocks). If the tumor is large enough (diameter > 10 mm), a sample is snap-frozen and stored at −80 °C in freezers located at each center. Frozen tumor samples are shipped every three months on dry ice to the biological resources platform of the Curie Institute in Paris for DNA, RNA and proteins extraction, aliquoting and storage at −80 °C. A representative FFPE block and slides used for diagnosis are sent at room temperature to the Mondor hospital’s biological resources platform for pathological review, DNA and RNA extraction, and Tissue Micro Arrays construction for future immunohistochemical and/or in situ hybridization analysis.

Cryosections are performed from frozen samples to check for tumor cell content by an uropathologist. Following the uropathologist report, macrodissections is performed on the frozen samples using a scalpel to take fragments enriched in tumor cells and the percentages of tumor cells of the microdissected fragments are recorded. When the samples are of sufficient size, a fragment of around 120 mg is taken. The remaining of the sample is stored at −80 °C. To extract RNA, DNA and protein from the same part of the tumor, each fragment is frozen in liquid nitrogen and is ground in a mortar. If the fragment is of sufficient size (above 45 mg), the powder is divided in three equal parts, for RNA and DNA extractions and for protein extract. If the quantity of material is limiting, DNA, then RNA are prioritized. Between 20 and 45 mg, the powder will be divided in two parts for RNA and DNA extractions. Below 20 mg, only DNA will be extracted.

DNA is extracted following a phenol-chloroform procedure and quantified using a NanoDrop spectrophotometer and a Qubit fluorometer. The quality of the DNA is verified by electrophoresis. RNA is extracted using the TissueLyzer (Qiagen) and the Qiagen miRNeasy kit. RNA is quantified with a Nanodrop spectrophotometer. The RIN (RNA integrity number) is measured on an Agilent 2100 bioanalyzer. For protein, the powder is dissolved in a Laemmli buffer using the TissueLyzer (Qiagen) and the proteins are quantified with the Pierce BCA kit. To spare materials, amplifications of RNA and DNA are performed as some analyses can be done on amplified materials. To obtain DNA and RNA from all tumors of the COBLAnCE cohort, DNA and RNA are being extracted from paraffin materials.

For FFPE samples, slides are prepared from each block. New HES coloration, as control, is realized, the slide is scanned and archived. A GATA3/CK5/6 double-staining immunohistochemistry is performed to classify the tumor at molecular level [13, 14] and guide Tissue Micro Arrays and DNA/RNA extraction performed on selected zones.

DNA and RNA (Recover All) are extracted using Maxwel automate and specific FFPE kits (Promega). DNA and RNA concentrations are quantified using Qubit technology. Samples are stored at −80 °C.

In case of recurrence, both fresh and paraffin-embedded tumor tissue samples are collected and processed as previously described.

#### Urine samples

A urine sample is collected prior to surgery. 50 ml of these urine samples is stored at room temperature in Norgen® tubes (Thorold, Canada) for nucleic acid extraction and the surplus is immediately frozen at −80 °C in Falcon tubes for further protein extraction.

Norgen® and frozen urine samples are shipped every three months at room temperature and on dry ice respectively to the Platform of Biological Resources - Henri Mondor Hospital to be processed.

DNA and RNA are extracted with the automated Maxwell 16 Instrument (Promega, Lyon, France), using the Maxwell LEV DNA Blood Kit and LEV RNA tissue Kit respectively. 25 ml of Norgen® urine is used for each DNA or RNA extraction on pellet after centrifugation. For DNA extraction, each sample is centrifuged at 2000 g for 10 min. Purified DNA and RNA samples are eluted in 50 μL nuclease-free water and stored at −80 °C in the Plateform of Biological Resources (Henri Mondor Hospital) until processed. Nucleic acids concentration is determined using NanoDrop 2000C (Thermo Scientific).

To extract urine protein, thawed urine is centrifuged at 1300 xg for 10 min at room temperature. Then, protein is isolated from both the supernatant and the obtained pellet separately. Proteins from the pellet are extracted by 200 μl of T-PER reagent (Tissue Protein Extraction Reagent, Thermo scientific), containing protease and phosphatase inhibitors. Proteins from the initial urine supernatant are precipitated by trichloracetic acid (TCA 30 %, Sigma-Aldrich) to a final concentration of 6 %, and the pellet is washed twice with ice-cold acetone (Sigma-Aldrich) and air-dried. Proteins are stored at −80 °C in the Platform of Biological Resources (Henri Mondor Hospital).

Protein concentration is assessed with Pierce BCA protein assay kit (Thermo Scientific).

An additional urine sample is collected at the 12 months follow-up visit for patients not treated with radical cystectomy and processed as previously described.

#### Toenail clippings

Toenail clippings are collected at the time the baseline questionnaire is completed and blood samples are collected. They are delivered to HPSC-BRC in a sealed envelope along with blood samples, and then mailed to the Mondor hospital’s biological resources platform every three months for storage at ambient temperature.

### Power considerations and data analysis

We planned to enroll 2,000 patients. We assumed a recruitment period of 5 years (50 % of the patients recruited the 3 first years and 50 % the 2 last years), a loss to follow-up rate of 10 % during the 3 first years, 5 % during the 2 following years, and 5 % thereafter, and a progression rate of 40 % at 4 years and 50 % at 5 years. Based on the literature data, 30 % of bladder cancer patients present with a MIBC at the time of diagnosis. We therefore expect 600 patients with MIBC to be included; the progression free survival is the primary endpoint of interest for these patients. The study will be sufficiently powered (bilateral test, α = 0.05, 1-β ≥ 80 %) to detect a hazard ratio ≥ 2 considering a prevalence of marker progression between 10 % and 20 %, or a hazard ratio ≥ 1.5 for a prevalence marker between 30 % and 50 %.

Data analyses will be performed to complement specific objectives outlined above. Various parameters which are planned to be assessed have been summarized in Table 1. All analyses will be carried out using SAS version 9.1 (SAS Institute, Cary, NC, USA) and STATA (Version 13, Stata Corporation, Austin, Texas).

## Discussion

To date (March 2016), 13 centers are actively involved in subject recruitment and 1020 patients have already been enrolled in the study. Recruitment is expected to end in the first semester of 2017. This ambitious project will allow having one of the biggest cohorts of incident bladder tumors with a longest follow-up (minimum 6 years).

This study will provide us with considerable insight into the biology of bladder cancer and the mechanisms through which various factors may alter the prognosis. It should facilitate the identification and the cost-effectiveness of emerging biomarkers and targeted therapies in bladder cancer.

The multidisciplinary approach of this study will allow communication with other researchers and departments to share ideas and foster collaborations. In fact epidemiologists, clinicians, biologists, pathologists, health economists, researchers coming from various French institutions joined together to build this cohort. In addition, the interaction with many health centers will support the translation of our research findings into clinical practice.

During the follow-up period, new experimental drugs for bladder cancer treatment may be recorded. For the purpose of these registrations, data prospectively collected in COBLAnCE will be useful to describe the natural history of bladder tumor evolution in France.

In summary, the establishment of this cohort study, along with the wealth of epidemiological, clinicipathological, economical and biological evidence to be collected, will lead to a much needed understanding of bladder cancer and provide evidence to guide health professionals in the management and care of patients with this disease.

Despite the large size of this prospective cohort, larger studies are necessary for specific analyses in subgroups of patients, i.e. within stage of disease, or within groups of patients with the same treatment. Therefore, we intend to establish close collaborations with other ongoing prospective studies on bladder cancer in order to combine the datasets and accordingly to increase the statistical power.

## Abbreviations

BRC: Biological resources center
eCRF: Electronic case report form
FFPE: Formalin-fixed paraffin-embedded
GSTM1: Glutathione-S-transferase M1
MIBC: Muscle-invasive bladder tumors
NAT2: N-acetyltransferase 2
NMIBC: Non-muscle-invasive bladder cancers
PBML: Peripheral blood mononuclear leukocytes
QoL: Quality of life
